# Risk perception, well-being, depression and anxiety in children and adolescents with rheumatic diseases during the COVID-19 pandemic - results from the prospective multicenter KICK-COVID study in Germany

**DOI:** 10.1186/s12969-024-00979-z

**Published:** 2024-04-18

**Authors:** Claudia Sengler, Jens Klotsche, Malthe Jessen Pedersen, Martina Niewerth, Julia Göldel, Daniel Windschall, Johannes-Peter Haas, Frank Dressler, Ralf Trauzeddel, Anton Hospach, Frank Weller-Heinemann, Stefanie Lanzinger, Clemens Kamrath, Reinhard W. Holl, Petra Warschburger, Kirsten Minden

**Affiliations:** 1 Deutsches Rheuma-Forschungszentrum Berlin, a Leibniz Institute, Epidemiology Unit, Charitéplatz 1, 10117 Berlin, Germany; 2 Department of Public Health, Aarhus University, Aarhus, Denmark; 3 Department of Psychology, Counselling Psychology, University of Potsdam, Potsdam, Germany; 4 Clinic for Pediatric and Adolescent Rheumatology, Northwest German Center for Rheumatology, St. Josef Stift Sendenhorst, Germany; 5 University of Halle-Wittenberg, Halle (Saale), Germany; 6 German Centre for Child and Adolescent Rheumatology, Pediatric Rheumatology, Garmisch-Partenkirchen, Germany; 7 Children’s Hospital, Hannover Medical School, Clinic for Pediatric Pneumology, Allergology and NeonatologyHannover Medical School, Hannover, Germany; 8 Department of Pediatrics, Pediatric and Adolescent Rheumatology, Helios Klinik Berlin-Buch, Berlin, Germany; 9 Department of Pediatrics, Olgahospital, Stuttgart, Germany; 10 Klinikum Bremen-Mitte, Eltern-Kind-Zentrum Prof. Hess, Pediatric Rheumatology, Bremen, Germany; 11 Institute of Epidemiology and Medical Biometry, ZIBMT, Ulm University, Ulm, Germany; 12 University Childrens Hospital, Giessen, Germany; 13 Department of Pediatric Respiratory Medicine, Immunology and Critical Care Medicine, Charité– Universitätsmedizin Berlin, corporate member of Freie Universität Berlin and Humboldt-Universität zu Berlin, Berlin, Germany

**Keywords:** COVID-19, Children, Adolescents, Rheumatic diseases, Risk perception, Well-being, WHO-5, PHQ-9, GAD-7

## Abstract

**Objective:**

To investigate the psychosocial burden in children and adolescents with juvenile rheumatic diseases during the COVID-19 pandemic.

**Methods:**

As part of the multicentre observational KICK-COVID study linked to the National Pediatric Rheumatology Database, adolescents < 21 years and parents of children < 12 years with rheumatic diseases answered questions on perceptions of health risk (PHR) due to SARS-CoV2, stress, well-being (WHO-5) and symptoms of depression (PHQ-9) and anxiety (GAD-7). Data were collected at routine visits from June to December 2021 and assessed for association with demographic and clinical parameters, treatment and patient-reported outcomes by multivariable regression analyses.

**Results:**

Data from 1356 individuals (69% female, 50% adolescents) were included. Median PHR on a numeric rating scale (NRS, 0–10) was 4 (IQR 2–6), median perceived stress was 3 (IQR 1–6). Adolescents reported a worse well-being with a significantly lower median WHO-5-score (60, IQR 40–76) than parents reported for their children < 12 years (80, IQR 68–84). Moderate to severe symptoms of depression and anxiety were reported by 14.3% and 12.3% of the adolescents, respectively. PHR was significantly higher in patients with systemic lupus erythematosus, methotrexate or biologic disease-modifying anti-rheumatic drug therapy than in patients without these characteristics, whereas lower WHO-5 or higher PHQ-9 or GAD-7 scores were only associated with poorer patient-reported health status and physical functioning.

**Conclusion:**

The perception of health risk due to SARS-CoV2 infection was not paralleled by an impairment of mental health, which were, however, significantly correlated with self-rated health status and functional capacity, highlighting the importance of patient-reported outcome assessment.

**Trial registration:**

German Clinical Trials Register (DRKS), no. DRKS00027974. Registered on 27th of January 2022.

**Supplementary Information:**

The online version contains supplementary material available at 10.1186/s12969-024-00979-z.

## Background

The pandemic of Corona virus disease 2019 (COVID-19) caused by severe acute respiratory syndrome corona virus type 2 (SARS-CoV-2) and the measures adopted to contain it significantly changed the daily lives of children and young people, both in terms of school attendance and leisure activities. Structural changes in daily routines (e.g. no school attendance) and limited opportunities to pursue hobbies or meet friends were already stressful for healthy people in terms of their psychosocial well-being [1]. Several studies published before the COVID-19 pandemic showed that children and adolescents with chronic diseases have a higher risk of developing mental health problems compared to their healthy peers [2, 3]. In a study published in 2013, Bomba et al. [4] found higher mean Children’s Depression Inventory (CDI) scores in children and adolescents with JIA than in healthy, age- and gender-matched control subjects; in addition, 23.3% of JIA patients and 8.3% of control subjects had a total CDI score of more than 17, indicating a significant risk of depression. In the UK Juvenile Idiopathic Arthritis (JIA) Inception Cohort “Childhood Arthritis Prospective Study” (CAPS), Hanns et al. found a proportion of 14.7% of adolescent patients with JIA who reached the cut-off score of the Mood and Feelings Questionnaire (MFQ) for major depressive disorder [5].Possible stress factors added by the pandemic could include concerns about a potential impairment of medical care or an increased risk of severe COVID-19 due to the underlying disease [6]. In patients with chronic rheumatic diseases who require immunomodulatory or even immunosuppressive therapy, the extent of a health risk from SARS-CoV-2 infection was initially unclear, which may have contributed to an increased perception of risk and this in turn may be related to more depressive or anxiety symptoms. Some studies have been able to show an increased psychosocial burden during the early phase of the COVID-19 pandemic (data from 2020) in young people and children with rheumatic diseases and their parents [7, 8]. The aim of our study was to investigate the psychosocial burden on children and adolescents with rheumatic diseases during the COVID-19 pandemic and to analyze possible influencing factors. Therefore, we (i) assessed the risk and stress perception, depression and anxiety symptoms, and general well-being in children and adolescents with rheumatic diseases and (ii) analysed the associations between demographic, clinical and therapeutic parameters and the above-mentioned outcomes in an advanced phase of the COVID-19 pandemic.

## Methods

### Patients and data source

The KICK-COVID study was launched to investigate the impact of the COVID-19 pandemic on health care, physical and mental health, and general well-being of children and adolescents with chronic diseases in Germany [9]. The multicenter prospective observational study assessed the short- and long-term consequences of the pandemic for children and adolescents with type 1 diabetes, obesity, and rheumatic diseases and their families. Recruitment and data collection was conducted through three established pediatric registries [10–12]. For rheumatic diseases like juvenile idiopathic arthritis (JIA), systemic lupus erythematosus (SLE), juvenile dermatomyositis (JDM), this was the National Pediatric Rheumatology Database (NPRD [12]): In this long-term observational study, children and adolescents with rheumatic diseases are included after informed consent (parents or children from the age of 8 years) and followed annually. Demographic and clinical parameters are recorded by the physician like age, sex, diagnosis, antinuclear antibody (ANA) positivity, age at disease onset, date of symptom onset and diagnosis, disease activity (NRS 0–10, 0 = no disease activity, NRS ≥ 1 = active disease) and joint count (only for JIA patients), and medication, specifically therapy with synthetic or biological disease-modifying antirheumatic drugs, sDMARD or bDMARD, respectively.

In order to be able to put the risk perception in the context of the actual hazard, information on a SARS-CoV-2 infection and its clinical course was collected in the NPRD. The following question with a yes/no-answer option was added to the physician’s questionnaire: “Has the patient ever been tested positive for SARS-CoV-2?” In case of a positive test, it was asked whether symptoms were present and whether hospitalization had occurred, also each with a yes/no-answer option.

In the NPRD, in addition to the treating physicians, patients 12 years of age and older and parents of children < 12 years of age were asked to provide information about their health or their child’s health on a NRS. The following patient-reported outcomes (PRO) were recorded: global assessment of health status on a numeric rating scale (NRS 0–10, 0 = best), pain (NRS 0–10, 0 = no pain) and functional status by the German version of the Childhood Health Assessment Questionnaire. The CHAQ assesses the ability to perform certain activities in 8 areas of daily life (dressing and personal hygiene, getting up, eating and drinking, walking, personal hygiene, reaching objects, grasping, activities and domestic tasks) using four response categories (easily possible = 0 points, slightly difficult = 1 point, very difficult = 2 points, not possible = 3 points). The item with the highest score per activity area is taken into account when calculating the total score. If help from other people or aids is required to carry out activities in an area, a score of at least 2 is awarded. The total score is then divided by 8 (number of areas of activity). The CHAQ score can therefore assume a value between 0 (no restriction) and 3 (maximum restriction).For further details please see [13]).

The assessments developed for the KICK-COVID study were collected as an add-on module to the patient/parent questionnaire alongside the other items in the NPRD. The institutions participating in the NPRD were asked to distribute the questionnaires to patients with rheumatic diseases 12 years or older or the parents of patients < 12 years of age during routine examinations. Completion of the KICK-COVID study questions was on a voluntary basis. For the analysis presented here, a subset of the questions (3 questions, see below) as well as scores on well-being, depressive as well as anxiety symptoms were evaluated. The data used were collected between June 2021 (start of implementation of the KICK-COVID questions in the NPRD) and December 2021, corresponding to the interim period (summer plateau 2021 [calendar week (CW) 24/2021-30/2021) and the fourth wave (CW 31/2021-51/2021; variant of concern (VOC) delta) of the SARS-CoV2 pandemic in Germany [14].

### Questions and questionnaires of the KICK-COVID study add-on module

#### Perception of health risk, stress and loneliness during the COVID-19 pandemic

The adolescents were asked how dangerous they consider a SARS-CoV2 infection to be for themselves.

For children with rheumatic diseases < 12 years of age, parents were asked how dangerous they consider a SARS-CoV2 infection to be for their child.

Perception of stress and loneliness were investigated by the question how stressed and how lonely young people with rheumatic diseases currently felt, respectively.

For children < 12 years, parents were asked to answer how stressed and how lonely their child currently felt, respectively.

The items were answered on a self-constructed 11-point numeric rating scale from 0 (“not dangerous/stressed/lonely at all”, respectively) to 10 (“totally dangerous/stressed/lonely”, respectively).

#### World Health Organization Five Well-Being Index (WHO-5)

The German version of the WHO-5 with 5 items was used to measure the general well-being of the participants in the last two weeks before their routine visits to the pediatric rheumatologist [15]. Responses were recorded using a 6-point Likert scale ranging from 0 “not at all” to 5 “always”. The WHO-5 raw score is calculated by summing up the numbers of the five answers and thus ranges from 0 (lack of well-being) to 25 (maximum well-being) and translated into a percentage scale from 0 to 100 by multiplying raw scores by 4, where 100 reflects best possible well-being. A WHO-5 score below 52 (raw score 13) indicates poor well-being and should lead to further psychological exploration [16]. The WHO-5 was filled out by the adolescents and for the children < 12 years by their parents on their behalf.

The WHO-5 has an internal consistency of Cronbach`s α = 0.89 to Cronbach`s α = 0.92 [15].

#### Patient Health Questionnaire-9 (PHQ-9)

The PHQ-9 consists of 9 questions related to symptoms of depression and was developed as a screening instrument. It assesses how often the respondent has shown depressive symptoms within the last 2 weeks [17] and has been validated for adolescents [18]. The answer categories are as follows: 0 (“Not at all”), 1 (“On some days”), 2 (“On more than half of the days”) and 3 (“Almost every day”). Accordingly, the sum of the scores is between 0 and 27, which are categorised as follows: 1–4: minimal depressive symptoms; 5–9: mild depressive symptoms; 10–14: moderate depressive symptoms; 15–27: severe depressive symptoms [17]. The PHQ-9 has an internal consistency of Cronbach`s α = 0.91 [19].

The PHQ-9 was only completed by patients ≥ 12 years.

#### Generalized anxiety disorder Scale-7 (GAD-7)

The GAD-7 consists of 7 questions that depict the core symptoms of a generalised anxiety disorder and measures the extent of generalised anxiety symptoms within the last 2 weeks [20]. It has also been validated for the use in adolescents [21].

There are the following response options: “not at all”, “on some days”, “on more than half of the days” and “almost every day”. These are assigned the numerical values 0 to 3, resulting in a sum of scores from 0 to 21, which are categorised as follows: 0–4: minimal anxiety symptoms, 5–9: mild anxiety symptoms, 10–14: moderate anxiety symptoms, 15–21: severe anxiety symptoms [20]. The GAD-7 has an internal consistency of Cronbach`s α = 0.79 to Cronbach`s α = 0.91 [22, 23]. The GAD-7 was only completed by patients ≥ 12 years.

### Statistics

Descriptive statistics were used to describe the distribution of categorical and continuously distributed parameters. Outcome parameters (perceived risk, stress, loneliness, WHO-5, PHQ-9, GAD-7) were compared by Mann-Whitney U-Test or Chi^2^-test between groups defined by disease parameters, sex, treatment and patient-reported health status. Multivariable linear regression analysis was performed in order to determine the association between the outcomes perceived health risk, stress, loneliness, WHO-5 score, PHQ-9 score and GAD-7 score with demographic, clinical, therapeutic and physician- as well as patient-reported outcome parameters (H). Standardized beta coefficients were calculated after fitting the linear regression models. As this was an exploratory study we did not adjust for multiple testing. All statistical analyses were performed with SAS 9.3 (SAS Institute Inc.).

## Results

Of 7278 patients documented from June to December 2021 by 49 centers participating in the NPRD, data from 1356 patients with KICK-COVID add-on module questionnaires (response rate 18.6%) from 674 adolescents < 21 years of age and 682 parents of children < 12 years of age with rheumatic diseases (JIA, SLE, JDM) could be analyzed. Demographic and clinical parameters, treatments and outcomes of the study cohort are reported in Table 1:

**Table 1:** Demographic and clinical parameter, treatment and outcome of children and adolescents in the study sample

Parameter	Patients ≥ 12 years	Patients < 12 years
*N*	674	682
Female subjects, n (%)	456 (67.7)	483 (70.8)
Age, years, mean (SD)	15.2 (1.9)	7.2 (2.9)
Age at disease onset, years, mean (SD)	8.8 (4.9)	4.3 (2.8)
Disease duration, years, mean (SD)	6.4 (4.9)	2.9 (2.7)
***Diagnoses, n (%)***		
JIA	620^1^	659^2^
Systemic arthritis	24 (3.6)	34 [5]
Oligoarthritis, extended	73 (10.8)	64 (9.4)
Oligoarthritis, persistent	214 (31.8)	355 (52)
Psoriatic arthritis	44 (6.5)	24 (3.5)
Enthesitis-related arthritis	85 (12.6)	19 (2.8)
Polyarthritis, seropositive	19 (2.8)	11 (1.6)
Polyarthritis, seronegative	127 (18.8)	124 (18.2)
Other arthritis	26 (3.9)	21 (3.1)
Systemic lupus erythematosus	42 (6.2)	6 (0.9)
Juvenile dermatomyositis	12 (1.8)	17 (2.5)
ANA positive/tested, n (%)	341/556; (61)	37/524; (71)
Therapy, n (%)*		
DMARD	352 (55.5)	346 (55)
sDMARD	262 (41.3)	290 (46.1)
bDMARD	195 (30.8)	124 (19.7)
Systemic glucocorticoids	35 (6.2)	24 (4.8)
***Outcome parameter***		
PhGA disease activity, NRS, mean (SD)	1.3 (1.8)	1.5 (2.2)
Joint count, mean (SD)^#^	1.1 (2.9)	1.2 (3.3)
Functional status, CHAQ (0–3), mean (SD)	0.2 (0.5)	0.3 (0.5)
PGA, NRS, mean (SD)	2.3 (2.5)	2.1 (2.5)

JIA: juvenile idiopathic arthritis; ANA: antinuclear antibodies; DMARD: disease modifying antirheumatic drugs; sDMARD: synthetic (conventional and targeted) disease modifying antirheumatic drugs; bDMARD: biologic disease modifying antirheumatic drugs; PhGA: physician`s global assessment of disease activity; PGA: Patient`s global assessment of health status *Percentages according to available valid information on the medication; ^#^only determined in JIA patients, *n* = 1279. ^1^There was a diagnosis of oligoarthritis without indication of persistent or extended in 5 cases, a diagnosis of polyarthritis without information on rheumatoid factor in 2 cases, and a diagnosis of JIA without indication of category in 1 case, which therefore could not be assigned. ^2^There was a diagnosis of oligoarthritis without indication of persistent or extended in 4 cases, a diagnosis of polyarthritis without information on rheumatoid factor in 1 case, and a diagnosis of JIA without indication of category in 2 cases

The distribution of diagnoses as well as the demographic, clinical, therapeutic and outcome parameters of the patients in our study cohort are largely comparable to the parameters of all patients documented in the NPRD from June to December 2021 (Additional file, Supplementary Table 2).

Questionnaires were completed broadly in equal numbers across the months from June 2021 to December 2021 (10–15% of the total per month), with July 2021 and October 2021 having proportionally the highest number of documentations (Table 2).

**Table 2:** Distribution of questionnaires in 2021, SARS Cov2 test results and overall incidence in Germany

Month of questionnaire administration	June 2021	July 2021	August 2021	September 2021	October2021	November 2021	December 2021
Questionnaires, n (%)	115 [10]	211 [18]	149 [13]	189 [16]	226 [19]	171 [14]	120 [10]
Patients, who reported to have ever been tested positive, n (%)	4 (3.5)	10 (4.7)	6 (4.0)	12 (6.3)	11 (4.9)	7 (4.1)	9 (7.5)
SARS-CoV2 incidence#	17	6	36	82	74	303	389

#infections per 100.000 inhabitants in Germany in the middle of the month [24]

Between the beginning of June 2021 and the end of October 2021, the measure index in Germany, that served as a measure of the type and number of restraints or restrictions on public life for infection control reasons, ranged between 40 and 30 (0 = no measure, 100 = most stringent measures), i.e. in the medium-low range, and then rose to an average of about 45 in November and to about 55 in December [25]

In the overall 1309 physician questionnaires, information on testing for SARS-CoV2 was provided for 1181 patients: 59 patients were reported to had ever been tested positive for SARS-CoV2 (5%) of whom 30 patients had developed symptoms (51%) and 2 patients had been hospitalised for COVID-19 (3%).

### Perception of health risk, stress and loneliness, well-being, symptoms of depression and anxiety

**Fig. 1:**
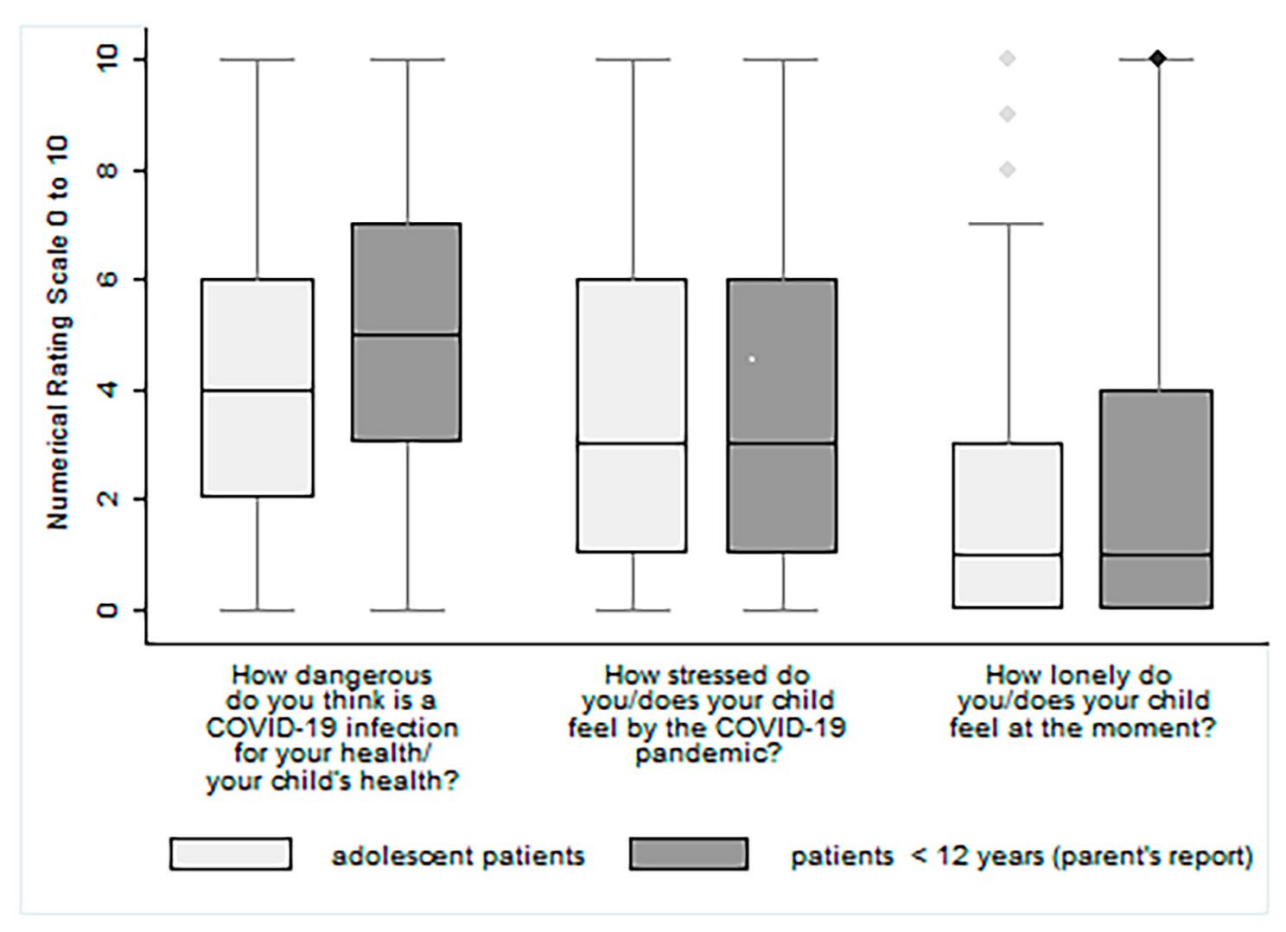
Box plots of answers to health-risk perception, stress and loneliness on a self-constructed 11-point NRS **Legend**: Shown are median values with interquartile ranges, stratified by age groups; 0: “not dangerous/stressed/lonely at all”; 10: “totally dangerous/stressed/lonely”

Adolescents and parents of children < 12 years of age with rheumatic diseases rated the danger of a SARS-CoV2 infection to their own or their children`s health with a median value of 4 and 5, respectively (Fig. 1).

The perception of stress related to the COVID-19 pandemic was reported by both adolescents and parents reporting for their children with a median value of 3, and the responses showed a wide range (IQR 1–6). The feeling of loneliness was rated by both groups with a median value of 1 (IQR 0–3, and IQR 0–4, respectively).

Overall, the WHO-5 score was lower in adolescents (mean 57.38 ± 22.97, median 60, IQR 40–76) than in children under 12 years of age (73.87 ± 19.11, median 80, IQR 68–84). A conspicuous WHO 5 score < 52 (raw score < 13) was reported by 36.5% of adolescent patients, compared with 11.5% of children younger than 12 years by their parents.

The mean PHQ-9 score of the adolescents was 4.5 ± 4.7 (median 3, IQR 1–7), the mean GAD-7 score was 4.2 ± 4.2 (median 3, IQR 1–6). A PHQ-9 score ≥ 10 was found in 14.2% and a GAD-7 score ≥ 10 in 12.3% of the adolescent patients.

### Association of outcome parameters (WHO-5, PHQ-9, GAD-7, perceived risk, stress and loneliness) with age, sex, disease activity, treatment, physician- and patient-reported outcome parameters

#### Univariable analysis

Among adolescents, female patients were found to have significantly lower WHO-5 scores, higher PHQ-9 scores and GAD-7 scores as well as a higher perception of SARS-CoV2-related health risk and loneliness compared to male patients. Among children < 12 years of age (parent-reported), girls had significantly higher mean WHO-5 scores than boys.

In addition, physician-reported active disease (NRS > 1) and at least one active joint (joint count ≥ 1) were significantly associated with a lower WHO-5 score. No significant association of WHO-5 score, PHQ-9 score or GAD-7 score (in adolescents) was found for therapy (no DMARD versus DMARD). However, the DMARD-treated patient groups reported significantly higher health risk perception than the patients without DMARD therapy.

Patient-reported impaired health status (PGA NRS > 1) and functioning (CHAQ > 0) were associated not only with a significantly lower WHO-5 score, higher PHQ-9 score and GAD-7 score (in adolescents) but also with higher perception of health risk, stress and loneliness. (Additional file, Supplementary Table 3) [26].

#### Multivariable analysis (data of adolescent patients)

Risk perception among adolescents was significantly associated with each of the following parameters: diagnosis of SLE, treatment with methotrexate, bDMARD therapy, PGA and physical functioning. Perceptions of stress and loneliness in adolescents were associated with female gender and again with patient’s assessment of health status and functioning (Table 3).

**Table 3:** Association of demographic, clinical, therapeutic parameters and PROs with health risk perception, stress and loneliness

	How dangerous…				How stressed…				How lonely…			
	beta	95% CI	*p* value	beta_ST_	beta	95% CI	*p* value	beta_ST_	beta	95% CI	*p* value	beta_ST_
Female	0.20	-0.27; 0.66	0.403	0.03	0.13	-0.40; 0.66	0.625	0.02	0.66	0.22; 1.10	0.003	0.12
Age	-0.02	-0.13; 0.10	0.742	-0,01	0.09	-0.04; 0.22	0.174	0.06	0.00	-0.11; 0.10	0.935	0.01
JIA	(ref)				(ref)				(ref)			
SLE	1.84	1.00; 2.68	< 0.001	0.18	0.49	-0.49; 1.46	0.328	0.04	0.02	-0.78; 0.82	0.953	0.01
JDM	0.37	-1.25; 2.00	0.651	0.02	0.02	-1.86; 1.91	0.980	0.01	-1.13	-2.68; 0.42	0.151	-0.06
PhGA	-0.14	-0.27; 0.00	0.054	-0.08	-0.03	-0.19; 0.13	0.732	-0.02	0.05	-0.08; 0.18	0.453	0.03
MTX	0.92	0.44; 1.40	< 0.001	0.16	0.45	-0.10; 1.00	0.109	0.07	0.16	-0.29; 0.61	0.498	0.03
bDMARD	0.54	0.06; 1.01	0.027	0.09	-0.16	-0.71; 0.39	0.567	-0.02	-0.10	-0.55; 0.35	0.651	-0.02
PGA	0.17	0.07; 0.27	0.001	0.15	0.22	0.11; 0.34	< 0.001	0.18	0.10	0.01; 0.19	0.045	0.09
CHAQ	0.85	0.30; 1.40	0.002	0.14	0.75	0.13; 1.38	0.019	0.11	1.04	0.52; 1.55	< 0.001	0.19

betaST: standardized beta coefficient; JIA: Juvenile idiopathic arthritis; SLE: Systemic lupus erythematosus; JDM: Juvenile dermatomyositis; PhGA: Physician`s global assessment of disease activity; MTX: Methotrexate; bDMARD: Biologic disease modifying antirheumatic drug; PGA: Patient`s global assessment of health status; CHAQ: Child health assessment questionnaire

Results of the multivariable analysis of these parameters in patients < 12 years of age (parents’ reports) are available in the supplement.

In addition, in adolescents there were significant associations of a lower WHO-5 score and a higher PHQ-9 score and GAD-7 score with female sex, PGA and physical functioning, but not with diagnosis (JIA, SLE, JDM) or treatment. For the PHQ-9, there was a significant association with the age of the patients (Table 4).

**Table 4:** Association of demographic, clinical and therapeutic parameters and PROs with WHO-5, PHQ-9 and GAD-7 scores

	WHO-5 score				PHQ-9 score				GAD-7 score			
	beta	95% CI	*p* value	beta_ST_	beta	95% CI	*p* value	beta_ST_	beta	95% CI	*p* value	beta_ST_
Female	-4.17	-8.06; -0.28	0.036	-0.08	1.65	0.87; 2.43	< 0.001	0.17	1.52	0.79; 2.24	< 0.001	0.17
Age	-0.94	-1.89; 0.01	0.052	-0.08	0.20	0.01; 0.40	0.037	0.08	0.14	-0.04; 0.31	0.134	0.06
JIA	(ref)				(ref)				(ref)			
SLE	3.31	-3.88; 10.50	0.367	0.04	-0.41	-1.87; 1.05	0.580	-0.02	-0.21	-1.54; 1.13	0.761	-0.01
JDM	5.13	-7.77; 18.04	0.435	0.03	-0.64	-3.41; 2.13	0.650	-0.02	-0.25	-2.84; 2.35	0.853	-0.01
PhGA	-0.78	-1.95; 0.40	0.196	-0.06	-0.11	-0.36; 0.13	0.375	-0.04	0.00	-0.23; 0.22	0.984	-0.01
MTX	-2.04	-6.01; 1.94	0.315	-0.04	0.50	-0.30; 1.29	0.223	0.05	0.64	-0.10; 1.38	0.088	0.07
bDMARD	1.76	-2.24; 5.76	0.389	0.03	0.16	-0.64; 0.96	0.692	0.02	-0.08	-0.82; 0.66	0.826	-0.01
PGA	-3.05	-3.91; -2.19	< 0.001	-0.32	0.58	0.41; 0.75	< 0.001	0.30	0.43	0.27; 0.58	< 0.001	0.24
CHAQ	-5.33	-9.91; -0.75	0.023	-0.10	1.68	0.72; 2.64	0.001	0.16	1.65	0.81; 2.50	< 0.001	0.18

betaST: standardized beta coefficient; JIA: Juvenile idiopathic arthritis; SLE: Systemic lupus erythematosus; JDM: Juvenile dermatomyositis; PhGA: Physician`s global assessment of disease activity; MTX: Methotrexate; bDMARD: Biologic disease modifying antirheumatic drug; PGA: Patient`s global assessment of health status; CHAQ: Child health questionnaire

## Discussion

In our study, 1 in 7 adolescents with a rheumatic disease reported moderate to severe depressive symptoms, and 1 in 8 adolescent patients described moderate to severe anxiety symptoms. In addition, somewhat more than a third of the adolescents reported significantly reduced general well-being. A significantly lower WHO-5 score in both age groups and significantly higher PHQ-9 and GAD-7 scores in adolescents were associated with poorer assessment of general health and functioning by patients or their parents. In contrast, there was no significant association between physician-assessed disease activity, number of active joints, treatment or education and these scores. Adolescents with a diagnosis of SLE or MTX or bDMARD therapy reported a significantly higher perception of health risk due to SARS-CoV2 infection, whereas this diagnosis or these therapies were not associated with higher levels of depressive or anxiety symptoms or lower well-being.

Only just under 5% of all patients reported that they had ever been tested positive for SARS-CoV2. Since at the time of our survey, Germany’s schools were regularly testing pupils for SARS-CoV2 nationwide, this information appears valid. According to the data collected here, only half of the patients who tested positive in our cohort had symptoms. We do not have information on the severity of COVID-19 in this cohort, however only 2 patients (3%) were hospitalised for COVID-19, suggesting a mostly mild disease course. According to a data collection originating from the early phase of the COVID-19 pandemic in Germany, SARS-CoV-2 infection in children and adolescents with rheumatic diseases under various medications was usually mild and had a good outcome in the majority of cases [27]. In the KICK-COVID study, adolescents as well as parents of children < 12 years of age indicated the risk of such an infection for their health/their child`s health in the middle range. Thus, there is some indication that the perception of health risk due to a SARS-CoV2 infection in our study population is higher than the actual health risk of such an infection. However, using data from the registry for COVID-19 in adults with rheumatic diseases, it has been shown that indeed certain immunosuppressive therapies (glucocorticoids, rituximab, Janus kinase inhibitors) increase the risk for a severe course of SARS-CoV2 infection [28]. Therefore, it seems important to inform parents, children and adolescents with rheumatic diseases about the COVID-19 related risks of an immunomodulatory or immunosuppressive therapy in order to reduce fears on the one hand and to encourage reasonable precautions on the other hand.

In our study, 14.2% and 12.3% of adolescent patients had moderate to severe symptoms of depression or anxiety, respectively. In the type 1 diabetes cohort of the KICK-COVID study, 74 patients (11.9%) reported moderate to severe anxiety symptoms, and the reported level of depressive symptoms in this cohort was moderate to severe in 88 (14.1%) participants [29], so there seem to be no relevant differences in the rate of these psychological comorbidities between these two cohorts despite different underlying chronic conditions. Futhermore, Kamrath et al. [29] showed that anxiety and depressive symptoms of type 1 diabetes patients during the COVID-19 pandemic were comparable to the assessments in these patients before the pandemic.

A meta-analysis of psychiatric comorbidities in patients with an immune-mediated disease diagnosed in childhood [30] revealed a prevalence of anxiety disorders of 13% (95% confidence interval: 12–15%) and of depression of 20% (95% confidence interval: 15–26%) for rheumatic diseases based on ICD-10 code or psychiatrist assessment. However, the included pre-pandemic studies differed considerably with regard to various parameters such as age and gender distribution as well as type and duration of illness. In the British JIA inception cohort Childhood Arthritis Prospective Study (CAPS), Hanns et al investigated mental comorbidities using the Mood and Feelings Questionnaire (MFQ): In 102 adolescent patients with JIA and short disease duration (mean 5 months), 14.7% exceeded the cut-off value (≥ 27) for major depressive disorder [5]. Fair et al. found similar results for children and adolescents with longer established JIA (mean duration of illness 4.7 years) using the Patient-Reported Outcome Measurement Information System (PROMIS) pediatric depressive symptoms and anxiety t-scores: Fourteen (17%) and 13 patients (16%) had moderate depressive or anxious symptoms, respectively, and 1 patient (1%) had severe depressive or anxious symptoms, respectively [31]. Overall, we can conclude that the proportion of adolescents with depressive or anxious symptoms in our study was similar to that described before the COVID-19 pandemic in the literature cited above for adolescents with JIA, who accounted for the majority of adolescent patients (92%) in our cohort. Furthermore, we found higher levels of anxiety and depression and lower levels of social well-being in female adolescents than in male adolescents. This is a long-standing observation that has been made in both healthy adolescents [32–34] and adolescents with rheumatic diseases [5] before the pandemic, and therefore our finding may not be attributed to pandemic-related stress factors, which are perceived differently by female adolescents than by male adolescents.The WHO-5 score as a measure of general well-being was on average lower in adolescents (median 60, IQR 40–76) than in children under 12 years of age (parent`s report, median 80, IQR 68–84) in our study. In the type 1 diabetes cohort of the KICK-COVID study (688 adolescents, mean age 15.6 ± 2.1, 45.6% female), the median score of the WHO-5 questionnaire was 56 (IQR 44–72), which was lower than in a pre-COVID-19 survey of a comparable cohort from Germany [29]. Furthermore, in our cohort of patients with rheumatic diseases, 36.5% of adolescent patients and 11.5% of children under 12 years of age (as reported by parents) had a WHO-5 score of < 52, a threshold that should lead to further psychological assessment [15]. In a study published in 2018, Balasz et al. examined the characteristics and correlates of physical disorders, self-rated health, subjective well-being and anxiety in 11,230 adolescents aged 14–16 years from 11 countries [35]. 15% of the participants answered “Yes” to the question: ”Do you suffer from a chronic illness?” In these individuals, the mean WHO-5 score was 59.5 ± 18.7 (adolescents in our study: 57.4 ± 23) and significantly lower than the WHO-5 score of healthy individuals (63.5 ± 20.6). In addition, Balasz reported more pronounced differences in the mean WHO-5 score between those with a chronic disease with resulting functional limitations in everyday life versus individuals with a chronic disease without resulting limitations, who had a WHO-5 score comparable to healthy individuals. Finally, the largest difference in the WHO-5 score was seen in the study by Balasz et al. between individuals who assessed their health status as poor or very poor (WHO-5 score 44 ± 21.6) versus subjects with a self-reported fair, good or very good state of health (WHO-5 score 63.4 ± 18.7). Our study revealed similar results: A significantly lower WHO-5 score, a higher PHQ-9 score and a higher GAD-7 score was found in patients with a poorer assessment of health status by the patient him- or herself and reported functional limitations. In the multivariable analysis, physician-assessed disease activity was not correlated with the PHQ-9 score, the GAD-7 score or the WHO-5 score. Already before the COVID-19 pandemic (data collection in 2019), Fair et al. found comparable results with depressive symptoms being correlated with the subjectively perceived functional impairment in everyday life (CHAQ score), pain and stress, but not with gender, JIA category, illness duration or activity (cJADAS-10) [31]. Hanns et al. described a significant association of symptoms of anxiety disorder and depression in JIA patients with functional limitations, pain and physician’s global assessment of disease activity, but not with inflammatory markers or number of active joints, based on data analysed before 2020 [36]. These data may suggest that the same parameters are associated with impaired well-being, depressive or anxious symptoms before and during the pandemic.

Adolescents’ health risk perception due to a SARS-CoV2 infection was associated with a diagnosis of SLE, treatment with methotrexate as well as bDMARD therapy, but not with WHO-5 score, PHQ-9 score or GAD-7 score in patients with this diagnosis or treatment. Perceptions of stress and loneliness were significantly higher in both adolescents and patients < 12 years of age (parental report) who reported poorer health and functional impairment, but no association was found with diagnosis, physician assessment of disease activity or treatment. It can be speculated that these patients may have fewer coping resources and therefore experience greater psychosocial stress during a pandemic with the changed circumstances of daily life. On the other hand, those who feel stressed during the pandemic may also have more difficulty managing their condition, for example in terms of adhering to medication or implementing physiotherapy. PROs, particularly PGA and physical functioning assessed with the CHAQ, appear to be important correlates of perceptions of stressors and strain as well as well-being during the pandemic, confirming the data of Peng et al. [7], whereas an increased perception of risk in certain patient groups did not translate into poorer mental health.

A limitation of our study is the lack of a matched pre-pandemic control group. Furthermore, selection bias cannot be excluded. Because this is a paper-and-pencil survey, patients must have been on-site for a medical consultation with their treating rheumatologist to fill in the KICK-COVID questionnaires. Therefore, those who visited a doctor more frequently than others, for example because of a more active condition, more severe pain or functional limitations, may have been more likely to participate in KICK-COVID. On the other hand, it might have been those who were significantly impaired who did not show up for regular follow-up visits because they considered themselves to be more at risk for COVID-19 infection and its consequences and therefore avoided places such as physicians’ offices or hospitals/outpatient clinics to reduce the risk of infection there. In addition, it is possible that the willingness to answer the questions on the KICK-COVID study varied in the different patient groups, which could have led to biases. The fact that the distribution of diagnoses and the demographic, clinical, therapeutic and outcome parameters of the patients in our study cohort are largely comparable to the parameters of all patients enrolled in the NPRD during the analyzed study period is a strength of our study, as is the large number of patients. Furthermore, by combining clinical, demographic and treatment data from the NPRD with results from the KICK-COVID add-on module, a wide range of possible associations could be investigated.

## Conclusions

Our data suggest that although a good third of adolescents reported significantly reduced general well-being, there does not appear to be increased mental health comorbidity in our cohort, since depressive and anxiety symptoms were reported at about the same level as in pre-pandemic studies of adolescents with rheumatic diseases. The perception of health risk due to SARS-CoV2 infection is not paralleled by an impairment of mental health and well-being in children and adolescents with rheumatic diseases, at least in the advanced pandemic situation in the second half of 2021. As before the pandemic, depressive symptoms as well as anxiety symptoms and well-being were associated with the perceived health situation and functioning related to the chronic disease, both in children and adolescents. This underlines the importance of patient-reported outcomes to uncover potential needs and provide targeted support services to improve both physical and mental health in the routine care of chronically ill children and adolescents.

### Electronic supplementary material

Below is the link to the electronic supplementary material.

Additional file 1: **Supplementary Table 1**: Demographic and clinical parameters, treatment and outcome of children and adolescents with systemic lupus erythematosus or juvenile dermatomyositis in the study sample. **Supplementary Table 2**: Demographic and clinical parameter, treatment and outcome of patients who participated in KICK-COVID and those included in the NPRD without participation in KICK-COVID. **Supplementary Table 3**: Association of outcome parameters (perceived risk, stress, loneliness, WHO-5, PHQ-9, GAD-7) with disease parameters, sex, treatment and patient-reported health status stratified by age group

## Data Availability

The datasets used and/or analysed during the current study are available from the corresponding author on reasonable request.

## Abbreviations

COVID-19: Corona Virus Disease 19
PHR: Perception of Health Risk
WHO: World Health Organization
PHQ-9: Patient Health Questionnaire 9
GAD-7: Generalized anxiety disorder 7
NRS: Numeric Rating Scale
IQR: Interquartile Range
PRO: Patient Reported Outcomes
DRKS: Deutsches Register für klinische Studien (German Clinical Trials Register)
SARS-CoV-2: Severe acute respiratory syndrome corona virus type 2
JIA: Juvenile idiopathic arthritis
SLE: Systemic lupus erythematosus
JDM: Juvenile idiopathic arthritis
NPRD: National Pediatric Rheumatology Database
ANA: Antinuclear antibodies
sDMARD: Synthetic disease modifying antirheumatic drug
bDMARD: Biologic disease modifying antirheumatic drug
CHAQ: Childhood Health Assessment Questionnaire
VOC: Variant of concern
PGA: Patient’s global assessment of health status
PhGA: Physician’s global assessment of disease activity
CAPS: Childhood Arthritis Prospective Study
PROMIS: Patient-Reported Outcome Measurement Information System
cJADAS-10: Clinical Juvenile Arthritis Disease Activity Score
