# Supplementary material for: Risk perception, well-being, depression and anxiety in children and adolescents with rheumatic diseases during the COVID-19 pandemic - results from the prospective multicenter KICK-COVID study in Germany
Source: Pediatr Rheumatol Online J. 2024 Apr 18;22:44. doi: 10.1186/s12969-024-00979-z (PMC11025219; doi:10.1186/s12969-024-00979-z)
Supplement: Supplementary file 1 — Additional file 1: Supplementary Table 1: Demographic and clinical parameters, treatment and outcome of children and adolescents with systemic lupus erythematosus or juvenile dermatomyositis in the study sample. Supplementary Table 2: Demographic and clinical parameter, treatment and outcome of patients who participated in KICK-COVID and those included in the NPRD without participation in KICK-COVID. Supplementary Table 3: Association of outcome parameters (perceived risk, stress, loneliness, WHO-5, PHQ-9, GAD-7) with disease parameters, sex, treatment and patient-reported health status stratified by age group [file 12969_2024_979_MOESM1_ESM.docx]

**Additional file 1**

**Supplementary Table 1**: **Demographic and clinical parameters, treatment and outcome of children and adolescents with systemic lupus erythematosus or juvenile dermatomyositis in the study sample**

| Parameter | Patients with SLE | Patients with JDM |
| --- | --- | --- |
| N | 48 | 29 |
| Female subjects, n (%) | 40 (83.3%) | 15 (51.7%) |
| Age, years, mean (SD) | 14.8 (3.6) | 10.5 (3.3) |
| Age at disease onset, years, mean (SD) | 10.7 (5.1) | 7.5 (3.3) |
| Disease duration, years, mean (SD) | 4.0 (4.7) | 3.1 (2.9) |
| ***Therapy, n (%)**** | |  |
| DMARD | 39 (84.8%) | 23 (79.3%) |
| csDMARD | 39 (84.8%) | 23 (79.3%) |
| JAK inhibitors | 0 (0.0%) | 1 (3.5%) |
| bDMARD | 4 (8.7%) | 1 (3.5%) |
| Rituximab | 1 (2.6%) | 0 (0.0%) |
| Systemic glucocorticoids | 17 (38.6%) | 11 (42.3%) |
| ***Outcome parameter*** | |  |
| PhGA disease activity, NRS, mean (SD) | 1.4 (2.1) | 1.7 (1.8) |
| Functional status, CHAQ (0-3), mean (SD) | 0.3 (0.6) | 0.5 (0.6) |
| PGA, NRS, mean (SD) | 2.2 (2.6) | 2.3 (2.5) |
| Pain, NRS, mean (SD) | 1.8 (2.5) | 1.2 (1.8) |
| Fatigue, NRS, mean (SD) | 2.6 (2.9) | 1.0 (2.1) |

(cs)DMARD: (conventional synthetic) disease modifying anti-rheumatic drug, b: biologic

JAK: Januse kinase

PhGA: physician`s global assessment of disease activity

PGA: Patient`s global assessment of health status

CHAQ: Childhood Health Assessment Questionnaire

*Percentages according to available valid information on medication (without missings)

**Supplementary Table 2: Demographic and clinical parameter, treatment and outcome of patients who participated in KICK-COVID and those included in the NPRD without participation KICK-COVID**

|  | Patients ≥ 12 years | | | Patients < 12 years | | |
| --- | --- | --- | --- | --- | --- | --- |
| Parameter | KickCOVID | NPRD w/o KICK-COVID | p | KICK-COVID | NPRD w/o KICK-COVID | p |
| N | 674 | 3492 |  | 682 | 2430 |  |
| Female subjects, n (%) | 456 (67.7) | 2,316 (66.6) | n.s. | 483 (70.8) | 1,712 (70.6) | n.s. |
| Age, years, mean (SD) | 15.2 (1.9) | 15.5 (2.5) | n.s. | 7.2 (2.9) | 7.3 (2.7) | n.s. |
| Age at disease onset, years, mean (SD) | 8.8 (4.9) | 8.7 (4.6) | n.s. | 4.3 (2.8) | 3.8 (2.6) | n.s. |
| Disease duration, years, mean (SD) | 6.4 (4.9) | 6.8 (4.6) | n.s. | 2.9 (2.7) | 3.5 (2.6) | n.s. |
| ***Diagnoses, n (%)*** | | | | | | |
| JIA | 620 | 3297 |  | 659 | 2357 | n.s. |
| Systemic arthritis | 24 (3.6) | 135 (3.9) | n.s. | 34 (5.0) | 110 (4.5) | n.s. |
| Oligoarthritis, extended | 73 (10.8) | 404 (11.6) | n.s. | 64 (9.4) | 223 (9.2) | n.s. |
| Oligoarthritis, persistent | 214 (31.8) | 979 (28.0) | n.s. | 355 (52.1) | 1,168 (48.0) | n.s. |
| Psoriatic arthritis | 44 (6.5) | 229 (6.6) | n.s. | 24 (3.5) | 104 (4.3) | n.s. |
| Enthesitis-related arthritis | 85 (12.6) | 641 (18.4) | 0.003 | 19 (2.8) | 88 (3.6) | n.s. |
| Polyarthritis, seropositive | 19 (2.8) | 105 (3.0) | n.s. | 11 (1.6) | 16 (0.7) | n.s. |
| Polyarthritis, seronegative | 127 (18.8) | 679 (19.4) | n.s. | 124 (18.2) | 524 (21.5) | n.s. |
| Other arthritis | 26 (3.9) | 101 (3.2) | n.s. | 21 (3.1) | 89 (3.7) | n.s. |
| Systemic lupus erythematosus | 42 (6.2) | 135 (3.9) | 0.005 | 6 (0.9) | 22 (0.9) | n.s. |
| Juvenile dermatomyositis | 12 (1.8) | 60 (1.7) | n.s. | 17 (2.5) | 51 (2.1) | n.s. |
| ANA positive/tested, n (%) | 341 (61.3) | 1,443 (58.4) | n.s. | 373 (71.2) | 1,423 (74.5) | n.s. |
| ***Therapy, n (%)**** | | | | | | |
| DMARD | 352 (55.5) | 2,114 (61.6) | 0.005 | 346 (55) | 1,464 (61.8) | 0.005 |
| sDMARD | 262 (41.3) | 1,444 (42.1) | n.s. | 290 (46.1) | 1,199 (50.6) | n.s. |
| bDMARD | 195 (30.8) | 1,251 (36.4) | 0.019 | 124 (19.7) | 647 (27.3) | 0.004 |
| Systemic glucocorticoids | 35 (6.0) | 178 (5.8) | n.s. | 24 (4.1) | 115 (5.4) | n.s. |
| ***Outcome parameter*** | | | | | | |
| PhGA disease activity, NRS, mean (SD) | 1.3 (1.8) | 1.1 (1.8) | n.s. | 1.5 (2.2) | 1.1 (1.8) | n.s. |
| Joint count, mean (SD)^#^ | 1.1 (2.9) | 0.8 (2.6) | n.s. | 1.2 (3.3) | 0.9 (2.8) | n.s. |
| Functional status, CHAQ (0-3), mean(SD) | 0.2 (0.5) | 0.2 (0.4) | n.s. | 0.3 (0.5) | 0.3 (0.5) | n.s. |
| PGA, NRS, mean (SD) | 2.3 (2.5) | 2.1 (2.4) | n.s. | 2.1 (2.5) | 1.4 (2.0) | n.s. |

**Supplementary Table 3: Association of outcome parameters (perceived risk, stress, loneliness, WHO-5, PHQ-9, GAD-7) with disease parameters, sex, treatment and patient-reported health status stratified by age group**

|  | **adolescent patients** | | | | **patients < 12 years (parents’ report)** | | | |
| --- | --- | --- | --- | --- | --- | --- | --- | --- |
|  | **Male**  **N = 218** | **Female**  **N = 456** | **d** | **p** | **Male**  **N = 199** | **Female**  **N = 483** | **d** | **p** |
| How dangerous… (0-10, 0 = totally harmless) | 3.9 (2.9) | 4.5 (2.6) | 0.18 | 0.037 | 4.8 (3.0) | 5.3 (2.7) | 0.09 | 0.046 |
| How stressed… (0-10, 0 = not stressed at all) | 3.6 (3.2) | 3.9 (2.9) | 0.10 | 0.268 | 3.2 (3.1) | 3.5 (3.0) | 0.04 | 0.319 |
| How lonely…(0-10, 0 = not lonely at all) | 1.4 (2.2) | 2.2 (2.7) | 0.31 | <0.001 | 2.0 (2.7) | 2.2 (2.7) | 0.03 | 0.471 |
| WHO-5-Score, mean (SD) | 61.2 (21.9) | 55.5 (23.2) | 0.25 | 0.003 | 71.2 (22.5) | 75.0 (18.4) | 0.10 | 0.031 |
| PHQ-9 Score, mean (SD) | 3.3 (4.0) | 5.1 (4.9) | 0.39 | <0.001 | - | - | - | - |
| GAD-7 Score, mean (SD) | 3.1 (3.8) | 4.7 (4.3) | 0.38 | <0.001 | - | - | - | - |
|  | **PhGA**  **NRS ≤ 1**  **N = 425** | **PhGA**  **NRS > 1**  **N = 223** | **d** | **p** | **PhGA**  **NRS ≤ 1**  **N = 450** | **PhGA**  **NRS > 1**  **N = 212** | **d** | **p** |
| How dangerous…(0-10, 0 = totally harmless) | 4.3 (2.7) | 4.3 (2.6) | 0.16 | 0.851 | 5.1 (2.8) | 5.1 (3.0) | 0.01 | 0.894 |
| How stressed…(0-10, 0 = not stressed at all) | 3.6 (3.0) | 4.3 (3.0) | 0.24 | 0.005 | 3.3 (2.9) | 3.7 (3.2) | 0.15 | 0.097 |
| How lonely…(0-10, 0 = not lonely at all) | 1.7 (2.5) | 2.2 (2.7) | 0.18 | 0.038 | 2.0 (2.7) | 2.3 (2.8) | 0.10 | 0.276 |
| WHO-5-Score | 60.9 (22.0) | 49.9 (23.6) | 0.49 | <0.001 | 76.8 (17.2) | 67.9 (21.3) | 0.47 | <0.001 |
| PHQ-9 Score, mean (SD) | 4.0 (4.7) | 5.3 (4.3) | 0.28 | 0.001 | - | - | - | - |
| GAD-7 Score, mean (SD) | 3.6 (4.0) | 5.0 (4.4) | 0.34 | <0.001 | - | - | - | - |
|  | **Joint count**  **= 0**  **N = 384** | **Joint count**  **≥ 1**  **N = 204** | **d** | **p** | **Joint count**  **= 0**  **N = 403** | **Joint count**  **≥ 1**  **N = 229** | **d** | **p** |
| How dangerous…(0-10, 0 = totally harmless) | 4.1 (2.7) | 4.3 (2.6) | 0.11 | 0.223 | 5.2 (2.8) | 5.0 (3.0) | 0.09 | 0.291 |
| How stressed…(0-10, 0 = not stressed at all) | 3.6 (2.9) | 4.0 (3.1) | 0.16 | 0.078 | 3.3 (2.8) | 3.5 (3.2) | 0.06 | 0.519 |
| How lonely…(0-10, 0 = not lonely at all) | 1.7 (2.3) | 2.2 (2.7) | 0.19 | 0.046 | 2.0 (2.6) | 2.2 (2.8) | 0.08 | 0.362 |
| WHO-5-Score | 60.1 (22.2) | 52.3 (23.5) | 0.34 | <0.001 | 77.0 (17.1) | 68.7 (21.1) | 0.44 | <0.001 |
| PHQ-9 Score, mean (SD) | 4.1 (4.8) | 4.9 (4.4) | 0.17 | 0.056 | - | - | - | - |
| GAD-7 Score, mean (SD) | 3.9 (4.2) | 4.7 (4.2) | 0.19 | 0.040 | - | - | - | - |
|  | **No DMARD**  **N = 290** | **DMARD**  **N = 366** | **d** | **p** | **No DMARD**  **N = 289** | **DMARD**  **N = 364** | **d** | **p** |
| How dangerous…(0-10, 0 = totally harmless) | 3.6 (2.5) | 4.8 (2.7) | 0.45 | <0.001 | 4.8 (2.8) | 5.4 (2.8) | 0.24 | 0.004 |
| How stressed…(0-10, 0 = not stressed at all) | 3.6 (2.9) | 3.9 (3.0) | 0.09 | 0.239 | 3.2 (2.9) | 3.6 (3.0) | 0.13 | 0.103 |
| How lonely…(0-10, 0 = not lonely at all) | 1.9 (2.5) | 1.9 (2.6) | 0.02 | 0.802 | 1.7 (2.4) | 2.4 (2.9) | 0.28 | <0.001 |
| WHO-5-Score | 58.3 (22.3) | 56.7 (23.7) | 0.07 | 0.418 | 75.3 (19.5) | 70.9 (20.8) | 0.19 | 0.021 |
| PHQ-9 Score, mean (SD) | 4.1 (4.6) | 4.7 (4.7) | 0.13 | 0.135 | - | - | - | - |
| GAD-7 Score, mean (SD) | 4.0 (4,2) | 4.3 (4.3) | 0.09 | 0.277 | - | - | - | - |
|  | **PGA**  **NRS ≤ 1**  **N = 301** | **PGA**  **NRS > 1**  **N = 349** | **d** | **p** | **PGA**  **NRS ≤ 1**  **N = 362** | **PGA**  **NRS > 1**  **N = 286** | **d** | **p** |
| How dangerous…(0-10, 0 = totally harmless) | 3.8 (2.7) | 4.7 (2.7) | 0.31 | <0.001 | 4.7 (2.8) | 5.6 (2.8) | 0.29 | <0.001 |
| How stressed…(0-10, 0 = not stressed at all) | 3.1 (2.9) | 4.3 (3.0) | 0.43 | <0.001 | 3.0 (2.8) | 3.8 (3.2) | 0.24 | 0.004 |
| How lonely…(0-10, 0 = not lonely at all) | 1.5 (2.3) | 2.2 (2.6) | 0.28 | <0.001 | 1.7 (2.5) | 2.5 (2.9) | 0.31 | <0.001 |
| WHO-5-Score | 66.4 (21.0) | 49.7 (21.8) | 0.78 | <0.001 | 80.2 (15.6) | 66.3 (19.7) | 0.79 | <0.001 |
| PHQ-9 Score, mean (SD) | 3.1 (4.2) | 5.7 (4.7) | 0.59 | <0.001 | - | - | - | - |
| GAD-7 Score, mean (SD) | 2.9 (3.8) | 5.2 (4.3) | 0.58 | <0.001 | - | - | - | - |
|  | **CHAQ = 0**  **N = 428** | **CHAQ > 0**  **N = 229** | **d** | **p** | **CHAQ = 0**  **N = 364** | **CHAQ > 0**  **N = 284** | **d** | **p** |
| How dangerous…(0-10, 0 = totally harmless) | 4.0 (2.7) | 4.8 (2.7) | 0.33 | <0.001 | 4.9 (2.7) | 5.4 (2.9) | 0.16 | 0.056 |
| How stressed…(0-10, 0 = not stressed at all) | 3.4 (2.9) | 4.5 (3.1) | 0.35 | <0.001 | 3.1 (2.7) | 3.8 (3.3) | 0.22 | 0.008 |
| How lonely…(0-10, 0 = not lonely at all) | 1.5 (2.2) | 2.5 (2.8) | 0.40 | <0.001 | 1.7 (2.4) | 2.7 (3.0) | 0.38 | <0.001 |
| WHO-5-Score | 62.2 (21.7) | 48.3 (23.0) | 0.63 | <0.001 | 79.5 (14.7) | 67.0 (21.5) | **0.69** | <0.001 |
| PHQ-9 Score, mean (SD) | 3.6 (4.4) | 6.3 (4.8) | 0.59 | <0.001 | - | - | - | - |
| GAD-7 Score, mean (SD) | 3.2 (3.7) | 6.0 (4.7) | 0.69 | <0.001 | - | - | - | - |
|  | **Grammar school**  **N = 248** | **Not at Grammar school**  **N = 326** | **d** | **p** | **Parents: University degree**  **N = 187** | **Parents: No univ. degree**  **N = 326** | **d** | **p** |
| How dangerous…(0-10, 0 = totally harmless) | 4.1 (2.4) | 4.5 (2.9) | 0.16 | 0.061 | 4.6 (2.6) | 5.2 (2.8) | 0.21 | 0.434 |
| How stressed…(0-10, 0 = not stressed at all) | 3.5 (2.6) | 4.0 (3.3) | 0.16 | 0.048 | 3.2 (2.8) | 3.4 (3.0) | 0.07 | 0.137 |
| How lonely…(0-10, 0 = not lonely at all) | 1.7 (2.3) | 2.1 (2.7) | 0.17 | 0.039 | 2.2 (2.7) | 2.1 (2.7) | 0.06 | 0.058 |
| WHO-5-Score | 57.9 (21.7) | 57.1 (23.5) | 0.03 | 0.691 | 74.5 (17.7) | 74.0 (19.2) | 0.03 | 0.088 |
| PHQ-9 Score, mean (SD) | 4.3 (4.5) | 4.6 (4.7) | 0.05 | 0.561 | - | - |  | - |
| GAD-7 Score, mean (SD) | 4.2 (4.2) | 4.1 (4.2) | 0.02 | 0.800 | - | - |  | - |

Values are mean (SD) if not otherwise stated; PhGA: Physician`s global assessment of disease activity; DMARD: Disease modifying antirheumatic drug; PGA: Patient`s global assessment of health status; CHAQ: Child health assessment questionnaire; WHO-5: World Health Organization Five Well-Being Index. d = Cohen’s d as a measure of effect size (26), interpretation: small (d = 0.2), medium (d = 0.5), and large (d = 0.8)

The complete questions about danger, stress and loneliness were:

“How dangerous do you think a SARS-CoV2 infection is for your health/your child’s health? (0-10, 0 = totally harmless)”

“How stressed do you/does your child feel by the COVID-19 pandemic? (0-10, 0 = not stressed at all)”

“How lonely do you/does your child feel at the moment? (0-10, 0 = not lonely at all)”
